# Cytenamide trifluoro­acetic acid solvate

**DOI:** 10.1107/S1600536808016577

**Published:** 2008-06-07

**Authors:** Andrea Johnston, Alastair J. Florence, Francesca J. A. Fabbiani, Kenneth Shankland, Colin T. Bedford, Julie Bardin

**Affiliations:** a Solid-State Research Group, Strathclyde Institute of Pharmacy and Biomedical Sciences, The John Arbuthnott Building, University of Strathclyde, 27 Taylor Street, Glasgow G4 0NR, Scotland; b University of Göttingen, GZG, Department of Crystallography, Goldschmidtstrasse 1, D-37077 Göttingen, Germany; c ISIS Facility, Rutherford Appleton Laboratory, Chilton, Didcot, Oxon OX11 0QX, England; d University College London, Department of Chemistry, 20 Gordon Street, London, WC1H 0AJ, England

## Abstract

Cytenamide forms a 1:1 solvate with trifluoro­acetic acid (systematic name: 5*H*-dibenzo[*a*,*d*]cyclo­hepta­triene-5-carboxamide trifluoro­acetic acid solvate), C_16_H_13_NO·C_2_HF_3_O_2_. The compound crystallizes with one mol­ecule of cytenamide and one of trifluoro­acetic acid in the asymmetric unit; these are linked by O—H⋯O and N—H⋯O hydrogen bonds to form an *R*_2_^2^(8) motif. The trifluoro­methyl group of the solvent mol­ecule displays rotational disorder over two sites, with site-occupancy factors of 0.964 (4) and 0.036 (4).

## Related literature

For details on the experimental methods used to obtain this form, see: Davis *et al.* (1964 ▶); Florence *et al.* (2003 ▶); Florence, Johnston, Fernandes *et al.* (2006 ▶). For literature on carbamazepine and other related structures, see: Cyr *et al.* (1987 ▶); Fleischman *et al.* (2003 ▶); Florence, Johnston, Price *et al.* (2006 ▶); Florence, Leech *et al*. (2006 ▶); Bandoli *et al.* (1992 ▶); Harrison *et al.* (2006 ▶); Leech *et al.* (2007 ▶); Florence, Bedford *et al.* (2008 ▶); Florence, Shankland *et al.* (2008 ▶); Fernandes *et al.* (2007 ▶). For hydrogen-bond motifs, see: Etter (1990 ▶); Bernstein *et al.* (1995 ▶).
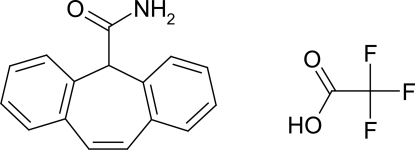


## Experimental

### 

#### Crystal data

C_16_H_13_NO·C_2_HF_3_O_2_
*M* *_r_* = 349.31
Monoclinic, 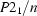

*a* = 12.1673 (11) Å
*b* = 6.3235 (6) Å
*c* = 21.4525 (15) Å
β = 101.932 (8)°
*V* = 1614.9 (2) Å^3^
*Z* = 4
Mo *K*α radiation
μ = 0.12 mm^−1^
*T* = 160 K
0.16 × 0.13 × 0.08 mm

#### Data collection

Oxford Diffraction Gemini S diffractometer
Absorption correction: multi-scan (*ABSPACK*/*CrysAlis RED*; Oxford Diffraction, 2006 ▶) *T*_min_ = 0.83, *T*_max_ = 0.99
10796 measured reflections
2995 independent reflections
1423 reflections with *I* > 2σ(*I*)
*R*_int_ = 0.094

#### Refinement

*R*[*F* ^2^ > 2σ(*F* ^2^)] = 0.080
*wR*(*F* ^2^) = 0.178
*S* = 1.04
2984 reflections
236 parameters
24 restraints
H-atom parameters not refined
Δρ_max_ = 0.73 e Å^−3^
Δρ_min_ = −0.60 e Å^−3^

### 

Data collection: *CrysAlis CCD* (Oxford Diffraction, 2006 ▶); cell refinement: *CrysAlis RED* (Oxford Diffraction, 2006 ▶); data reduction: *CrysAlis RED*; program(s) used to solve structure: *SHELXS97* (Sheldrick, 2008 ▶); program(s) used to refine structure: *CRYSTALS* (Betteridge *et al.*, 2003 ▶); molecular graphics: *ORTEP-3* (Farrugia, 1997 ▶) and *Mercury* (Macrae *et al.*, 2006 ▶); software used to prepare material for publication: *publCIF* (Westrip, 2008 ▶) and *PLATON* (Spek, 2003 ▶).

## Supplementary Material

Crystal structure: contains datablocks I. DOI: 10.1107/S1600536808016577/cf2202sup1.cif

Structure factors: contains datablocks I. DOI: 10.1107/S1600536808016577/cf2202Isup2.hkl

Additional supplementary materials:  crystallographic information; 3D view; checkCIF report

## Figures and Tables

**Table 1:** Hydrogen-bond geometry (Å, °)

*D*—H⋯*A*	*D*—H	H⋯*A*	*D*⋯*A*	*D*—H⋯*A*
O3—H5⋯O2^i^	0.89	1.58	2.462 (4)	173
N1—H11⋯O1^ii^	0.86	2.23	2.976 (4)	144
N1—H12⋯O1^iii^	0.87	2.16	2.982 (5)	159

Symmetry codes: (i) 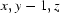; (ii) 
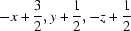
; (iii) 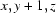.

**Fig. 1.**
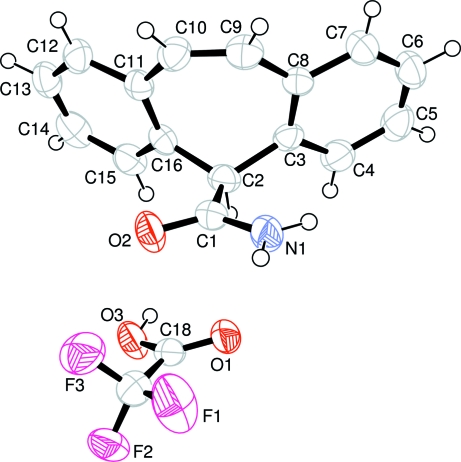
The molecular structure of CYT-TFAA, showing 50% probablility displacement ellipsoids. The lower occupancy fluorine atoms have beem omitted for clarity.

**Fig. 2.**
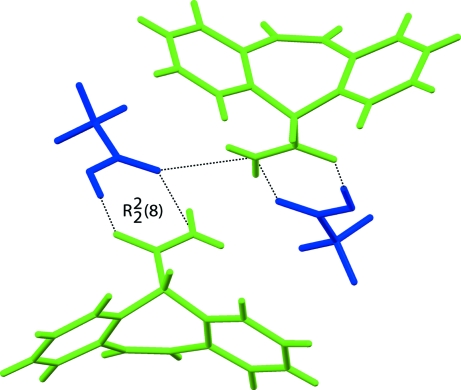
Hydrogen-bonded contacts in CYT-TFAA, showing the adjacent R22(8) CYT-TFAA units further linked by an N—H···O interaction. Minor ocupancy components have been omitted for clarity. CYT and TFAA molecules are coloured according to symmetry equivalence (green and blue respectively) and hydrogen bonds are shown as dashed lines.

## supplementary crystallographic information

### Comment

Cytenamide (CYT) is an analogue of carbamazepine (CBZ), a dibenzazepine drug used to control seizures (Cyr *et al.*, 1987). Cytenamide-trifluoroacetic acid solvate (CYT-TFAA) was produced during an automated parallel crystallization study (Florence, Johnston, Fernandes *et al.*, 2006) of CYT as part of a wider investigation that couples automated parallel crystallization with crystal structure prediction methodology to investigate the basic science underlying the solid-state diversity of CBZ (Florence, Johnston, Price *et al.*, 2006; Florence, Leech *et al.*, 2006) and its closely related analogues: CYT (Florence, Bedford *et al., *2008), 10,11-dihydrocarbamazepine (Bandoli *et al.*, 1992; Harrison *et al.*, 2006; Leech *et al.*, 2007) and cyheptamide (Florence, Shankland *et al.*, 2008). The sample was identified as a new form using multi-sample foil transmission X-ray powder diffraction analysis (Florence *et al.*, 2003). Subsequent manual recrystallization from a saturated TFAA solution by slow evaporation at 278 K yielded a sample suitable for single-crystal X-ray diffraction (Fig. 1).

The compound crystallizes in space group *P*2_1_/n with one molecule of CBZ and one molecule of TFAA in the asymmetric unit. As in the structure of CBZ-TFAA solvate (Fernandes *et al.*, 2007) the solvent molecule displays rotational disorder and the fluorine atoms were refined over two sites yielding site occupancy factors 0.964 (4), 0.036 (4) and 0.53 (1), 0.47 (1) for CYT-TFAA and CBZ-TFAA respectively. The molecules also adopt a hydrogen-bonded arrangement similar to that observed in CBZ-TFAA solvate whereby the amide group of each CYT molecule is connected to the carboxylic acid group of a TFAA molecule by N–H···O and O–H···O contacts (Table 1) to form an *R*_2_^2^(8) hydrogen-bonded motif (Etter, 1990; Bernstein *et al.*, 1995). CYT also forms a second N—H···O contact with an adjacent solvent molecule to form a chain extending along the [010] direction.

### Experimental

A sample of cytenamide was synthesized according to a modification of the published method (Davis *et al.*, 1964). A single-crystal sample of cytenamide-TFAA was grown from a saturated TFAA solution by isothermal solvent evaporation at 278 K.

### Refinement

Owing to the weak scattering, data were integrated applying a theta cut off of 25°. All non-hydrogen atoms were modelled with anisotropic displacement parameters with the exception of the minor component of the disordered site in the TFAA molecule, for which one common isotropic displacement parameter was calculated and fixed during refinement. Bond-length restraints were applied to C—F bond lengths involving atoms F1, F4, F5 and F6. 3-Fold symmetry was imposed on the disordered minor site of the the TFAA molecule by the use of restraints. H-atoms were found in a difference Fourier map and were initially refined with soft restraints on the bond lengths and angles to regularize their geometry and *U*_iso_(H) (in the range 1.2–1.5 times *U*_eq_ of the parent atom), after which the positions were fixed. Eleven reflections were suppressed as outliers in an analysis of the data.

### Crystal data

**Table:**

C_16_H_13_NO·C_2_HF_3_O_2_	*F*_000_ = 720
*M**_r_* = 349.31	*D*_x_ = 1.437 Mg m^−^^3^
Monoclinic, *P*2_1_/*n*	Mo *K*α radiation λ = 0.71073 Å
Hall symbol: -P 2yn	Cell parameters from 1137 reflections
*a* = 12.1673 (11) Å	θ = 3–25º
*b* = 6.3235 (6) Å	µ = 0.12 mm^−^^1^
*c* = 21.4525 (15) Å	*T* = 160 K
β = 101.932 (8)º	Block, colourless
*V* = 1614.9 (2) Å^3^	0.16 × 0.13 × 0.08 mm
*Z* = 4	

### Data collection

**Table:**

Oxford Diffraction Gemini S diffractometer	2995 independent reflections
Radiation source: Enhance (Mo) X-ray Source	1423 reflections with *I* > 2σ(*I*)
Monochromator: graphite	*R*_int_ = 0.094
Detector resolution: 15.9745 pixels mm^-1^	θ_max_ = 25.5º
*T* = 160 K	θ_min_ = 3.1º
φ and ω scans	*h* = −14→14
Absorption correction: multi-scan(ABSPACK/CrysAlis RED; Oxford Diffraction, 2006)	*k* = 0→7
*T*_min_ = 0.83, *T*_max_ = 0.99	*l* = 0→25
10796 measured reflections	

### Refinement

**Table:**

Refinement on *F*^2^	Primary atom site location: structure-invariant direct methods
Least-squares matrix: full	Hydrogen site location: inferred from neighbouring sites
*R*[*F*^2^ > 2σ(*F*^2^)] = 0.080	H-atom parameters not refined
*wR*(*F*^2^) = 0.178	Method = Modified Sheldrick *w* = 1/[σ^2^(*F*^2^) + ( 0.06*P*)^2^ + 0.42*P*] ,where *P* = (max(*F*_o_^2^,0) + 2*F*_c_^2^)/3
*S* = 1.05	(Δ/σ)_max_ = 0.0004
2984 reflections	Δρ_max_ = 0.73 e Å^−^^3^
236 parameters	Δρ_min_ = −0.60 e Å^−^^3^
24 restraints	Extinction correction: None

### Fractional atomic coordinates and isotropic or equivalent isotropic displacement parameters (Å^2^)

**Table:**

	*x*	*y*	*z*	*U*_iso_*/*U*_eq_	Occ. (<1)
C1	0.6284 (3)	0.6720 (7)	0.3296 (2)	0.0461	
C2	0.5488 (4)	0.4854 (7)	0.31163 (18)	0.0431	
C3	0.4806 (3)	0.4971 (7)	0.24379 (18)	0.0380	
C4	0.4965 (4)	0.3481 (7)	0.1996 (2)	0.0507	
C5	0.4406 (4)	0.3618 (8)	0.1366 (2)	0.0578	
C6	0.3726 (4)	0.5332 (9)	0.1180 (2)	0.0576	
C7	0.3553 (4)	0.6826 (8)	0.16104 (19)	0.0519	
C8	0.4077 (3)	0.6646 (7)	0.22567 (17)	0.0393	
C9	0.3794 (4)	0.8214 (7)	0.2691 (2)	0.0475	
C10	0.3773 (3)	0.7968 (7)	0.33147 (19)	0.0456	
C11	0.4028 (3)	0.6095 (7)	0.37105 (18)	0.0369	
C12	0.3488 (4)	0.5854 (7)	0.42250 (19)	0.0470	
C13	0.3646 (4)	0.4094 (8)	0.45999 (18)	0.0515	
C14	0.4337 (4)	0.2524 (8)	0.4474 (2)	0.0570	
C15	0.4912 (4)	0.2755 (7)	0.3988 (2)	0.0497	
C16	0.4774 (3)	0.4536 (7)	0.36104 (17)	0.0374	
C17	0.8909 (4)	0.3112 (8)	0.4387 (2)	0.0572	
C18	0.8197 (3)	0.1386 (7)	0.4008 (2)	0.0461	
N1	0.6679 (3)	0.7697 (6)	0.28446 (15)	0.0533	
O1	0.7936 (2)	0.1490 (5)	0.34338 (13)	0.0564	
O2	0.6593 (2)	0.7215 (5)	0.38684 (12)	0.0591	
O3	0.7904 (3)	−0.0019 (5)	0.43735 (12)	0.0618	
F1	0.9217 (4)	0.4524 (6)	0.40116 (15)	0.1040	0.964 (4)
F2	0.9828 (2)	0.2331 (5)	0.47540 (13)	0.0714	0.964 (4)
F3	0.8361 (3)	0.4082 (5)	0.47792 (16)	0.0845	0.964 (4)
F4	0.846 (3)	0.502 (2)	0.429 (3)	0.0800*	0.036 (4)
F5	0.992 (2)	0.330 (8)	0.425 (3)	0.0800*	0.036 (4)
F6	0.910 (5)	0.282 (6)	0.5010 (4)	0.0800*	0.036 (4)
H11	0.6461	0.7332	0.2450	0.0619*	
H12	0.7150	0.8730	0.2945	0.0619*	
H21	0.5969	0.3601	0.3139	0.0495*	
H41	0.5449	0.2331	0.2125	0.0596*	
H51	0.4499	0.2559	0.1077	0.0680*	
H61	0.3386	0.5487	0.0754	0.0651*	
H71	0.3068	0.7953	0.1473	0.0581*	
H91	0.3579	0.9563	0.2521	0.0539*	
H101	0.3556	0.9169	0.3516	0.0541*	
H121	0.3022	0.6959	0.4312	0.0539*	
H131	0.3278	0.3947	0.4943	0.0619*	
H141	0.4423	0.1260	0.4713	0.0647*	
H151	0.5414	0.1676	0.3914	0.0557*	
H5	0.7404	−0.0935	0.4169	0.0888*	

### Atomic displacement parameters (Å^2^)

**Table:**

	*U*^11^	*U*^22^	*U*^33^	*U*^12^	*U*^13^	*U*^23^
C1	0.033 (2)	0.065 (3)	0.040 (3)	−0.002 (2)	0.005 (2)	0.003 (2)
C2	0.036 (2)	0.047 (3)	0.044 (2)	0.008 (2)	0.002 (2)	−0.006 (2)
C3	0.028 (2)	0.046 (3)	0.041 (2)	−0.007 (2)	0.0095 (19)	−0.007 (2)
C4	0.044 (3)	0.056 (3)	0.052 (3)	0.000 (2)	0.011 (2)	−0.009 (2)
C5	0.056 (3)	0.072 (4)	0.048 (3)	−0.012 (3)	0.015 (2)	−0.021 (3)
C6	0.053 (3)	0.081 (4)	0.036 (3)	−0.011 (3)	0.002 (2)	−0.003 (3)
C7	0.044 (3)	0.070 (3)	0.041 (3)	−0.004 (2)	0.004 (2)	0.002 (3)
C8	0.030 (2)	0.049 (3)	0.038 (2)	−0.003 (2)	0.0052 (19)	0.000 (2)
C9	0.047 (3)	0.041 (3)	0.055 (3)	0.002 (2)	0.009 (2)	−0.001 (2)
C10	0.047 (3)	0.045 (3)	0.047 (3)	0.003 (2)	0.013 (2)	−0.006 (2)
C11	0.036 (2)	0.037 (3)	0.035 (2)	−0.006 (2)	0.0022 (19)	−0.006 (2)
C12	0.047 (3)	0.056 (3)	0.038 (2)	−0.005 (2)	0.008 (2)	−0.011 (2)
C13	0.050 (3)	0.069 (4)	0.035 (2)	−0.014 (3)	0.007 (2)	0.006 (3)
C14	0.058 (3)	0.057 (3)	0.050 (3)	−0.010 (3)	−0.004 (2)	0.011 (3)
C15	0.043 (3)	0.055 (3)	0.048 (3)	−0.003 (2)	0.001 (2)	0.002 (2)
C16	0.040 (3)	0.036 (3)	0.032 (2)	−0.002 (2)	−0.0011 (19)	−0.003 (2)
C17	0.054 (3)	0.064 (4)	0.055 (3)	−0.003 (3)	0.014 (3)	−0.002 (3)
C18	0.034 (3)	0.060 (3)	0.044 (3)	0.002 (2)	0.007 (2)	0.008 (3)
N1	0.043 (2)	0.078 (3)	0.038 (2)	−0.017 (2)	0.0045 (17)	−0.009 (2)
O1	0.0488 (19)	0.082 (2)	0.0375 (17)	−0.0082 (17)	0.0064 (14)	0.0091 (17)
O2	0.054 (2)	0.090 (3)	0.0300 (17)	−0.0283 (18)	0.0024 (14)	−0.0002 (16)
O3	0.060 (2)	0.085 (2)	0.0362 (17)	−0.0297 (19)	−0.0009 (15)	0.0067 (17)
F1	0.132 (3)	0.101 (3)	0.074 (2)	−0.059 (3)	0.008 (2)	0.020 (2)
F2	0.0448 (18)	0.091 (2)	0.0715 (19)	−0.0066 (16)	−0.0045 (14)	−0.0160 (17)
F3	0.075 (2)	0.086 (2)	0.095 (2)	0.0090 (19)	0.0230 (19)	−0.027 (2)

### Geometric parameters (Å, °)

**Table:**

C1—C2	1.525 (6)	C11—C16	1.386 (5)
C1—N1	1.321 (5)	C12—C13	1.363 (6)
C1—O2	1.247 (4)	C12—H121	0.942
C2—C3	1.521 (5)	C13—C14	1.364 (6)
C2—C16	1.517 (6)	C13—H131	0.942
C2—H21	0.980	C14—C15	1.378 (6)
C3—C4	1.379 (6)	C14—H141	0.945
C3—C8	1.384 (5)	C15—C16	1.376 (5)
C4—C5	1.383 (6)	C15—H151	0.951
C4—H41	0.941	C17—C18	1.520 (5)
C5—C6	1.372 (7)	C17—F1	1.307 (4)
C5—H51	0.936	C17—F2	1.322 (5)
C6—C7	1.368 (6)	C17—F3	1.328 (5)
C6—H61	0.928	C17—C18	1.520 (5)
C7—C8	1.406 (5)	C17—F4	1.323 (7)
C7—H71	0.934	C17—F5	1.323 (7)
C8—C9	1.451 (5)	C17—F6	1.323 (7)
C9—C10	1.352 (5)	C18—O1	1.209 (4)
C9—H91	0.944	C18—O3	1.283 (5)
C10—C11	1.453 (6)	N1—H11	0.865
C10—H101	0.938	N1—H12	0.867
C11—C12	1.405 (5)	O3—H5	0.887
			
C2—C1—N1	119.0 (4)	C12—C11—C16	118.2 (4)
C2—C1—O2	119.3 (4)	C11—C12—C13	121.4 (4)
N1—C1—O2	121.6 (4)	C11—C12—H121	118.3
C1—C2—C3	113.4 (4)	C13—C12—H121	120.4
C1—C2—C16	110.5 (3)	C12—C13—C14	119.6 (4)
C3—C2—C16	113.4 (3)	C12—C13—H131	120.7
C1—C2—H21	105.8	C14—C13—H131	119.7
C3—C2—H21	106.9	C13—C14—C15	120.2 (4)
C16—C2—H21	106.2	C13—C14—H141	120.7
C2—C3—C4	119.9 (4)	C15—C14—H141	119.0
C2—C3—C8	119.8 (4)	C14—C15—C16	120.8 (4)
C4—C3—C8	120.2 (4)	C14—C15—H151	119.6
C3—C4—C5	121.2 (4)	C16—C15—H151	119.6
C3—C4—H41	119.6	C2—C16—C11	120.1 (4)
C5—C4—H41	119.2	C2—C16—C15	120.1 (4)
C4—C5—C6	118.6 (4)	C11—C16—C15	119.7 (4)
C4—C5—H51	120.0	C18—C17—F1	111.5 (4)
C6—C5—H51	121.3	C18—C17—F2	111.6 (4)
C5—C6—C7	121.1 (4)	F1—C17—F2	107.9 (4)
C5—C6—H61	119.4	C18—C17—F3	111.4 (4)
C7—C6—H61	119.5	F1—C17—F3	108.7 (4)
C6—C7—C8	120.6 (4)	F2—C17—F3	105.6 (4)
C6—C7—H71	119.3	C18—C17—F4	113.56 (6)
C8—C7—H71	120.1	C18—C17—F5	113.57 (6)
C7—C8—C3	118.2 (4)	F4—C17—F5	105.08 (7)
C7—C8—C9	117.4 (4)	C18—C17—F6	113.57 (6)
C3—C8—C9	124.4 (4)	F4—C17—F6	105.08 (7)
C8—C9—C10	127.8 (4)	F5—C17—F6	105.08 (7)
C8—C9—H91	116.8	C17—C18—O1	120.4 (4)
C10—C9—H91	115.3	C17—C18—O3	111.8 (4)
C9—C10—C11	128.8 (4)	O1—C18—O3	127.8 (4)
C9—C10—H101	115.3	C1—N1—H11	120.6
C11—C10—H101	115.9	C1—N1—H12	119.5
C10—C11—C12	118.0 (4)	H11—N1—H12	119.9
C10—C11—C16	123.9 (4)	C18—O3—H5	113.5

### Hydrogen-bond geometry (Å, °)

**Table:**

*D*—H···*A*	*D*—H	H···*A*	*D*···*A*	*D*—H···*A*
O3—H5···O2^i^	0.89	1.58	2.462 (4)	173
N1—H11···O1^ii^	0.86	2.23	2.976 (4)	144
N1—H12···O1^iii^	0.87	2.16	2.982 (5)	159

Symmetry codes: (i) *x*, *y*−1, *z*; (ii) −*x*+3/2, *y*+1/2, −*z*+1/2; (iii) *x*, *y*+1, *z*.
